# An Economic Model of Human Cooperation Based on Indirect Reciprocity and Its Implication on Environmental Protection

**DOI:** 10.3390/ijerph15071303

**Published:** 2018-06-21

**Authors:** Jugui Dai, Yiqiang Zhang, Victor Shi

**Affiliations:** 1 School of Economics, Zhejiang University, Hangzhou 310027, China; daijugui@163.com; 2 College of Business, Wenzhou University, Wenzhou 325035, China; 3 Xulun Honor School, Shanghai Lixin University of Accounting and Finance, Shanghai 201209, China; 4 School of Business and Economics, Wilfrid Laurier University, Waterloo, ON N2N1E9, Canada; cshi@wlu.ca

**Keywords:** human cooperation, altruism behavior, indirect reciprocity, game

## Abstract

There has been an urgent challenge for environmental protection due to issues like population increase, climate change, and pollution. To address this challenge, sustained human cooperation is critical. However, how cooperation in human beings evolves is one of the 125 most challenging scientific questions, as announced by *Science* in its 125th anniversary. In this paper, we contribute to answering this question by building an economic game model based on indirect reciprocity and altruism behavior. In our model, there are three types of participants: cooperator, defector, and discriminator. In every round of the game, the cooperator chooses cooperation, the defector chooses non-cooperation, and the choice of the discriminator depends on the choice of his partner in the last round. Our analysis and main result shows that there is no stable evolution equilibrium in this game, which implies that the proportions of different types of players will keep changing instead of reaching a stable equilibrium. In other words, there is no guarantee that cooperation will be dominant in this game. An implication of this result is that to achieve cooperation and protect the environment more effectively, cooperators and discriminators in our society should be provided with incentives.

## 1. Introduction

Since the seventeenth century, mankind’s thinking has been profoundly affected by the views of survival of the fittest by Darwin in natural selection and rational egoism in economics. With fierce competition in the economic, political, military, and other areas since the industrial revolutions, especially after the brutal World War I and World War II, competition has become the main focus of people’s thinking. Moreover, Bentham’s utilitarianism and Hobbes’s Law of the Jungle have become the philosophical basis of the competition concept. However, biologists find that there are altruistic behaviors in many species. Recent research also indicates that if there is no cooperation in ancient times, the anthropoid ape could not evolve into human beings, and human beings also could not reach the top of the food chain today. Consequently, it is very important to explore the origin and the evolution of cooperation of human beings. *Science* has announced 125 most challenging scientific problems, among which “how cooperation in human beings evolves” is one of the two major problems in social science that will drive the basic scientific research and determine the future direction of scientific research in the next hundred years [1]. Many researchers are interested in cooperation of human beings, including anthropologists, sociologists, economists, biologists, and neuroscientists. These researchers aim to reveal the contributing factors to human cooperation from different perspectives.

Classical economics explains altruistic behavior of human beings from ethics. David Hume argues that human’s morals and emotions come from sympathy [2]. Adam Smith analyzed the origin of sympathy and its different manifestations. He used ethics as the basis of the origin of human cooperation and altruistic behavior. However, ethics itself is also a result of cooperative altruism. Based on the hypothesis of rational egoism, neoclassical economics derives a comprehensive theoretical system from self-interested individual behavior by deductive approach. Neoclassical economics argues that self-interested individual is willing to sacrifice short-term personal interests in order to benefit more in the future, which leads to cooperative altruistic behavior. The Reputation Theory is such an example. Nonetheless, the Cooperation Theory of neoclassical economics based on self-interests cannot explain the numerous instances of pure altruism, i.e., sacrifice without compensation. Based on the rational self-interested hypothesis, the subgame perfect Nash equilibrium leads to the conclusion that playing “prisoner’s dilemma” for finite times cannot engender cooperative behavior.

Fortunately, there are still biologists who rescue us from the moral dilemma of economics. Biological research finds that the altruistic behavior is one of the important factors to promote cooperation and improve competitiveness in the early stage of human beings. Biologists divide altruistic behavior into kin altruism, reciprocal altruism, and pure altruism. Reciprocal altruism, which can be divided into direct reciprocal altruism and indirect reciprocal altruism, is a kind of non-kin altruism. Direct reciprocity refers to the mechanism in which the donor gets favor from the recipient, i.e., “you scratch my back and I will scratch yours,” while indirect reciprocity refers to the mechanism in which the donor does not obtain favor from the recipient but from an irrelevant third party who can at the same time harvest good reputation. Alexander proposed the idea of indirect reciprocity for the first time in 1987 [3]. Many scholars believe that indirect reciprocity is the origin of human ethics, and the cooperation of human beings in the process of evolution is based on indirect reciprocity. The study of indirect reciprocity emerged from biology at first but has since been widely used in other disciplines, including sociology, economics and anthropology [4,5].

To help understand how cooperation in human beings evolves, in this paper, we build an economic game model with three types of players: the cooperator, the defector, and the discriminator. In each round of the game, the cooperator chooses cooperation and the defection chooses non-cooperation or defection. Starting with the second round, the discriminator chooses cooperation if his partner chooses cooperation in the last round and chooses defection if his partner chooses defection in the last round. Hence, the game is based on indirect reciprocity.

We model and analyze the human cooperation model based on indirect reciprocity in this paper partly because we aim to understand how we can cooperate more effectively to achieve environmental protection. It is well known that there have been a number of urgent environmental issues such as pollution and climate change. For example, of the 100 million metric tons of plastic produced every year, about 10 million end up in the oceans. In terms of electronic waste, about an average of 50 million tons is produced every year [6]. A big reason for this to happen is that a significant proportion of people and organizations have low regard for environmental protection. Furthermore, when they choose to litter or at least not to recycle, they face insufficient penalties. Admittedly, with a current world population of about 7.3 billion, it might be impossible for everyone not to be a defector in the game of environmental protection [7]. On the other hand, this research can shed a light on how the different types of players will evolve as far as environmental protection is concerned. In particular, we aim to provide argument for the importance for government and the society as a whole to provide incentives to people to take actions for environmental protection.

The rest of this paper is organized as follows. In Section 2, we provide a concise literature review. We then set up our main model and analyze it in Section 3. In Section 4, we extend our main model and show that the results from our main model are robust. Finally, in Section 4, we summarize our results and discuss their implications for environmental protection.

## 2. Literature Review

This paper focus on explaining human cooperative behavior using an economic game model based on indirect reciprocity and altruism behavior. In our review of the related literature, we first focus on research on kin-based altruism behavior, then on research on non-kin third-party altruism behavior. For an existing study to be included in our review, the study should have a focus on theoretical explanation of human altruism cooperation. Note that such a study may be from diverse research areas such as biology, neuroscience, and economics.

In nature, kin altruism is the most common type of altruism and is considered to be a kind of “innate preference” for the animals. Hamilton, a British biologist, is the first person who explained kin altruism behavior in biology utilizing game theory. However, he could not explain non-kin altruism in human society. Human beings are the only species in nature who have non-kin altruism behavior. What is the origin of non-kin altruism behavior and how does it evolve? Some scholars explain non-kin altruism behavior from biological evolution mechanisms. The famous Santa Fe School considers that the altruism behavior of early humans is the result of internalization of social norms, and the harsh natural environment forces humans to extend altruism from kin individuals to non-kin individuals in order to raise the species fitness [8]. Biological evolution mechanism includes group selection theory and individual selection theory. The group selection theory posits that natural selection mechanism occurs on the level of biological species. If it can improve a species’ fitness, then altruism behavior can stay with the evolution of that species group. [9] explains the altruism of human from the preference of prosocial other-regarding. They presume that when humans act, they consider not only the consequences for themselves (self-regarding) but also the consequences for others (other-regarding). The pressure of group selection impels human to have prosocial features. Neurophysiology finds that the rational selection of human beings is deeply affected by emotions such as sympathy and guilt. Taking the feelings of others into account, human beings will adjust their behavior. The individual selection theory holds that natural selection mechanisms only arises on the level of individuals. In other words, individuals only give consideration to their own interests in the process of evolution. In the famous book *The Selfish Gene*, Richard Dawkins points out that all living things are selfish in essence. Even if there is cooperation between them, the only reason is for more benefit in the future. [10] argues that the reason of altruism is the surplus obtained from cooperation. Even self-interested individuals will take altruistic actions in order to establish an efficient cooperative order and improve the competitive advantage of humans in the biological world.

There is strong reciprocal altruism among human non-kin altruistic behavior. Strong reciprocal altruism means that one takes action that is only beneficial for others but is costly for himself or herself. It is also called “altruistic punishment.” [11] explains the reason of altruistic punishment from the angle of group selection. They believe that human can build up a social order of cooperation and punishment by copying and learning the behavior of the majority and other successful people. [8] also points out that although the altruistic punishment will reduce the fitness of the punisher himself, other members of the group will benefit more if all of them comply with social norms and punish the violator. Some neuron economists think that there is a so-called “opium reward area” in human’s brain. When the area is stimulated, it will produce a self-incentive mechanism, which makes people act with strong reciprocal altruism.

Because of the cost of altruistic punishment, it is easy to lead to the “second-order free rider problem,” which can make the punishment mechanism fail [12]. In order to solve the dilemma of the second-order free rider, [13] proposes a coordination penalty model, which shows that if the number of individuals in a population conforming to social norms increases, and the cost of altruistic punishment decreases, then altruistic punishment behavior will be common in the population. [14] also argue that plenty of altruistic punishment behavior can overcome the problem of second-order free rider in the process of human evolution, and hence achieve human cooperation. [15] demonstrates by a public goods game that if the reward of public goods reaches a certain degree, stable punishment can be a permanent threat to defection and can effectively maintain the order of human cooperation. He also holds that social justice is the prerequisite to solve the social dilemma, and the sense of justice in human nature is the outcome of internalization of social norms.

Reciprocal altruism is another non-kin altruism, including direct reciprocal altruism and indirect reciprocal altruism. Direct reciprocity can explain the repeated cooperation game among non-kin players, but it cannot explain third-party reciprocal cooperation, i.e., indirect reciprocal cooperation. [16] challenges the traditional direct reciprocity model based on prisoner’s dilemma by setting up the first indirect reciprocity model. They incorporate reputation into their model and analyze its evolutionarily stable condition. [17] proposes that the moral signal in human social networks can boost indirect reciprocal cooperation. Some researchers investigated the stable evolutionary result of the standing strategy under the condition of indirect reciprocity [18]. [19] compares the evolutionary equilibria of three games: the prisoner dilemma, the stag hunt game, and the snowdrift game. They find that under the condition of indirect reciprocity, the influences of ethics, reputation, and other mechanisms on final evolutionary equilibrium can be very complicated depending on the initial game conditions. However, [20] finds that there is a need of a small number of defectors in a group to encourage other group members to punish the second-order free riders. This can promote cooperative behavior within the group. Therefore, the existence of bad actors is also valuable for the evolution of human, just as the moderate existence of native predators can eventually raise the fitness of a prey species.

In this paper, we set up and analyze a game of indirect reciprocity which includes three types of actors: cooperator, discriminator, and defector. In the game, the discriminator will punish the defector. We demonstrate that the game does not have a stable evolution equilibrium, which proves that there is also the “one-third law” in the evolution of indirect reciprocity. That is, in the process of human evolution, not only cooperators and discriminators but also defectors are needed. In order to further verify the effect of altruistic punishment, we extend our main model so that in the extended model, players can observe each other’s choices of the last two rounds. In this extended model, our analysis shows that the proportion of players who will choose cooperation increases starting from the third round. However, the payoffs of the cooperators and the defectors stay the same, while the payoff of the discriminators may or may not increase.

## 3. Main Model and Analysis

Suppose there are three types of players in a game, including the cooperator, the defector and the discriminator. In each round, a player’s choice can be observed by others, but the player can only remember the choice from the last round. The initial percentages of cooperator, defector, and discriminator in the population are $x_{1}$, $x_{2}$, and $x_{3}$, respectively, and it is common information. In every round, the cooperator chooses cooperation, and the defector chooses non-cooperation. The choice of discriminator depends on the choice of his partner in the last round. If the partner’s choice is cooperation in last round, then the discriminator chooses to cooperate in this round. If the partner’s choice is defection in the last round, the discriminator also chooses to defect in this round. If two players both choose to cooperate, they will obtain benefit π and incur cost c, where π>c. If one chooses to cooperate and the other chooses to defect, the cooperator’s benefit is v and cost is c , and the defector’s benefit also is v but cost is 0, where π>v. We assume that π is in proportion of v, i.e., v=θπ(0<θ<1). If two players choose noncooperation, their benefits and costs are zero. See Table 1 for a summary of the potential payoffs.

Before our detailed analysis, we summarize the basic steps in our analysis and the similarities and differences between our model and existing models reviewed in the previous section. As for the basic steps of our analysis, we first derive the total payoffs of the cooperator, the defector, and the discriminator. Based on these payoffs, we will obtain the dynamic changes of the proportions of these types of player. Then we investigate if there is a stable equilibrium point in this economic game. As can be seen in our following analysis, consistent with existing models reviewed in the literature, our model assume that a play can only observe another player’s choices of two rounds: the current round and the previous round. However, different from existing studies, our model explicitly obtains the dynamics of the proportions of the three player types and find that in the long term, there is no stable equilibrium point in this game.

A player only can observe his partner’s last round choice. In the first round, there is no information about the game, so it assumes that the proportion of discriminator who chooses cooperation in the first round is p. From the second round, the discriminator chooses cooperation if his partner chooses cooperation in the last round and chooses defection if his partner chooses defection in the last round. Hence, it is a downstream indirect reciprocity. Note that upstream indirect reciprocity and downstream indirect reciprocity are defined as follow. Supposing there are three players: A, B, and C. Upstream indirect reciprocity means if A helps B first, then B will help C next. Downstream indirect reciprocity means if A helps B first, then C will help B after observing B’s behavior.

We use $g_{n}$ to denote the proportion of players who will choose cooperation in the *n*th round. Then we have: $g_{1}=x_{1}+px_{3}$ and $g_{2}=x_{1}+g_{n-1}x_{3}(n\geq 2)$. Furthermore, we can obtain the following equation:

(1) $$g_{n}=\frac{x_{1}}{1-x_{3}}+{\left(x_{3}\right)}^{n}(p-\frac{x_{1}}{1-x_{3}})(n\geq 2)$$

In the first round, for the cooperator, the chance that his/her opponent cooperates is $x_{1}+px_{3}$, and the expected revenue is $\left(x_{1}+px_{3})(\pi -c\right)$. The chance that his/her opponent defects is 1 − $\left(x_{1}+px_{3}\right)$, and the expected revenue is $\left[1-(x_{1}+px_{3})](v-c\right)$. Hence, the cooperator’s expected revenue is

$$\begin{array}{l} (x_{1}+px_{3})(\pi -c)+[1-(x_{1}+px_{3})](\theta \pi -c)=(x_{1}+px_{3})\pi +[1-(x_{1}+px_{3})]\theta \pi -c\\ =-c+\theta \pi +(1-\theta )(x_{1}+px_{3})\pi \end{array}$$

where we use the relationship v=θπ in the derivation. Similarly, we can obtain that the discriminator’s revenue is $-cp+p\theta \pi +(p+\theta -2p\theta )(x_{1}+px_{3})\pi$ and the defector’s revenue is $(x_{1}+px_{3})\theta \pi$. In the *n*th round (n≥2), the cooperator’s revenue is $-c+\theta \pi +(x_{1}+x_{3})(1-\theta )\pi$, and the defector’s revenue is $x_{1}\theta \pi$. The proportion of the discriminator who chose cooperation in the last round is $g_{n-1}$, and his revenue in this round is $g_{n}(\pi -c)+(1-g_{n})(x_{1}+x_{3})\theta \pi$. The proportion of the discriminator who chooses noncooperation in the last round is $1-g_{n-1}$, and his revenue in this round is $g_{n}(\pi -c)+(1-g_{n})x_{1}\theta \pi$. Therefore, the discriminator’s expected revenue in this round is $g_{n-1}[g_{n}(\pi -c)+(1-g_{n})(x_{1}+x_{3})\theta \pi ]+(1-g_{n-1})[g_{n}(\pi -c)+(1-g_{n})x_{1}\theta \pi ]$. According to Equation (1), the discriminator’s expected revenue can be simplified as $g_{n}(1+\theta -\theta g_{n})\pi -cg_{n}(n\geq 2)$. At the end of the *n*th round, the total payoff of the cooperator, the defector and the discriminator will be as follows:

$$R_{1}=-Nc+N\theta \pi +N(1-\theta )(x_{1}+x_{3})\pi -(1-p)(1-\theta )x_{3}\pi ,$$

$$R_{2}=Nx_{1}\theta \pi +px_{3}\theta \pi ,$$

$$R_{3}=-cp+p\theta \pi +(p+\theta -2p\theta )(x_{1}+px_{3})\pi +(\pi +\theta \pi -c)\sum_{n=2}^{N}g_{n}-\theta \pi \sum_{n=2}^{N}{\left(g_{n}\right)}^{2}$$

where

$$\sum_{n=2}^{N}g_{n}=(N-1)\frac{x_{1}}{1-x_{3}}+(p-\frac{x_{1}}{1-x_{3}})\frac{{\left(x_{3}\right)}^{2}-{\left(x_{3}\right)}^{N+1}}{1-x_{3}},$$

$$\sum_{n=2}^{N}{\left(g_{n}\right)}^{2}=(N-1){\left(\frac{x_{1}}{1-x_{3}}\right)}^{2}+2\frac{x_{1}}{1-x_{3}}(p-\frac{x_{1}}{1-x_{3}})\frac{{\left(x_{3}\right)}^{2}-{\left(x_{3}\right)}^{N+1}}{1-x_{3}}+\frac{{\left(x_{3}\right)}^{4}-{\left(x_{3}\right)}^{2(N+1)}}{1-{\left(x_{3}\right)}^{2}}{\left(p-\frac{x_{1}}{1-x_{3}}\right)}^{2}$$

Let ${\overset{\wedge }{R}}_{1}=R_{1}-R_{2}$, ${\overset{\wedge }{R}}_{2}=R_{2}-R_{2}$, ${\overset{\wedge }{R}}_{3}=R_{3}-R_{2}$, then

$$\begin{array}{l} {\overset{\wedge }{R}}_{1}=-Nc+N\theta \pi +N(1-2\theta )x_{1}\pi +[N(1-\theta )+p+\theta -1-2p\theta ]x_{3}\pi ;\\ {\overset{\wedge }{R}}_{2}=0;\\ {\overset{\wedge }{R}}_{3}=-cp+p\theta \pi +(p+\theta -2p\theta -N\theta )x_{1}\pi +(1-2\theta )p^{2}x_{3}\pi +(\pi +\theta \pi -c)\sum_{n=2}^{N}g_{n}-\theta \pi \sum_{n=2}^{N}{\left(g_{n}\right)}^{2} \end{array}$$

The mean payoff of the three players is $\overset{-}{R}=x_{1}{\overset{\wedge }{R}}_{1}+x_{2}{\overset{\wedge }{R}}_{2}+x_{3}{\overset{\wedge }{R}}_{3}$. Substituting ${\overset{\wedge }{R}}_{1}$, ${\overset{\wedge }{R}}_{2}$, ${\overset{\wedge }{R}}_{3}$ into the equations, we have the following:

$$\begin{array}{l} \overset{-}{R}=-Ncx_{1}+[-cp+p\theta \pi +(\pi +\theta \pi -c)\sum_{n=2}^{N}g_{n}+\theta \pi \sum_{n=2}^{N}{\left(g_{n}\right)}^{2}]x_{3}+[N(1-2\theta )\\ +2p+2\theta -1-4p\theta ]x_{1}x_{3}\pi \end{array}$$

The dynamics of the cooperator, the defector and the discriminator are as follows:

$$\begin{array}{l} {\overset{•}{x}}_{1}=x_{1}({\overset{\wedge }{R}}_{1}-\overset{-}{R})=x_{1}[{\overset{\wedge }{R}}_{1}-(x_{1}{\overset{\wedge }{R}}_{1}+x_{3}{\overset{\wedge }{R}}_{3})]=x_{1}[(1-x_{1}){\overset{\wedge }{R}}_{1}-x_{3}{\overset{\wedge }{R}}_{3}];\\ {\overset{•}{x}}_{2}=x_{2}({\overset{\wedge }{R}}_{2}-\overset{-}{R})=-x_{2}(x_{1}{\overset{\wedge }{R}}_{1}+x_{3}{\overset{\wedge }{R}}_{3});\\ {\overset{•}{x}}_{3}=x_{3}({\overset{\wedge }{R}}_{3}-\overset{-}{R})=x_{3}[{\overset{\wedge }{R}}_{3}-(x_{1}{\overset{\wedge }{R}}_{1}+x_{3}{\overset{\wedge }{R}}_{3})]=x_{3}[(1-x_{3}){\overset{\wedge }{R}}_{3}-x_{1}{\overset{\wedge }{R}}_{1}]. \end{array}$$

${\overset{•}{x}}_{i}$ indicates the dynamic change of the proportions of the three player types as the game evolves. Solving the equations of ${\overset{\wedge }{R}}_{1}$ and ${\overset{\wedge }{R}}_{3}$, we can obtain the values of $x_{1}$ and $x_{3}$. According to $x_{1}+x_{2}+x_{3}=1$, we can then calculate $x_{2}$. In this way, we can calculate the stable equilibrium points. Because the equations are nonlinear and analytically not solvable, we use numerical simulation with Matlab to obtain $x_{1}$, $x_{2}$, and $x_{3}$.

In our simulations, we use the following values for the parameters: N=100, $\pi =10^{6}$, $c=0.5\times 10^{6}$, p=0.5, and θ=0.75. Employing Matlab programming, we obtain two solutions of $x_{1},x_{3}:(0.24,-0.52)$ and (0.665,0.3367). However, because of the requirements of $x_{1}+x_{2}+x_{3}=1$ and $x_{i}\in [0,1]$, there is no real solution in the interior of the definition domain, which means there is no stable equilibrium point. In other words, the dynamic system is chaotic. The conclusion conforms to the “one-third law,” which means that if there are three strategies in the evolution, those three strategies will be dominant in turn [21]. Bowles, S. et al also finds that there is no stable evolutionary equilibrium if a group consists of only cooperators [8]. A group will be extinct if the group consists of only defectors. However, a group that includes some discriminators can survive in the competition of evolution. Perhaps, as Kauffman stated, life exists on the edge of chaos. In the same logic, evolutionary equilibrium based on indirect reciprocity exists on the edge of chaos.

**Figure 1:**
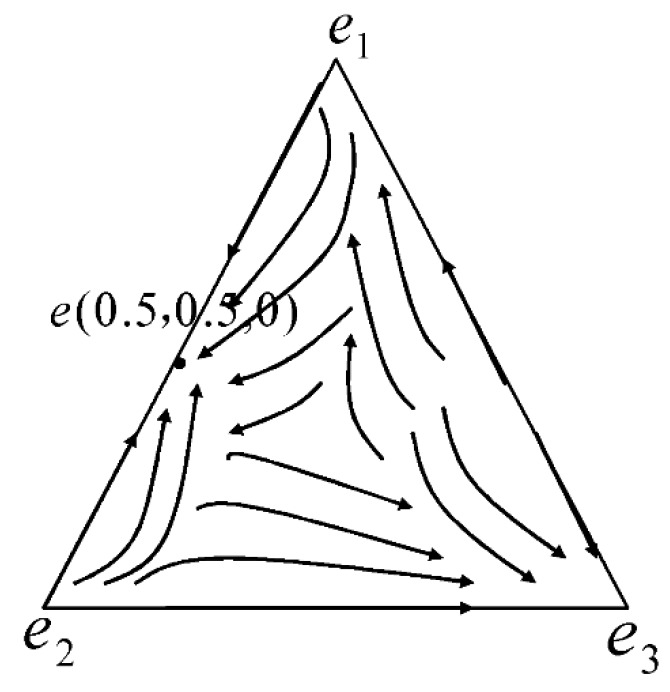
Phases of indirect reciprocity evolution.

In the absence of the cooperator, we have $x_{1}=0$, $R_{2}=R_{3}$, and ${\overset{\wedge }{x}}_{2}+{\overset{\wedge }{x}}_{3}=1$. And the stable equilibrium point is $\overset{\wedge }{e}(0,{\overset{\wedge }{x}}_{2},{\overset{\wedge }{x}}_{3})$. Using the following relationship,

$$p(2\theta -1)\pi x_{3}+p\theta \pi \frac{{\left(x_{3}\right)}^{4}-{\left(x_{3}\right)}^{2(N+1)}}{1-{\left(x_{3}\right)}^{2}}-(\pi +\theta \pi -c)\frac{{\left(x_{3}\right)}^{2}-{\left(x_{3}\right)}^{N+1}}{1-x_{3}}=\theta \pi -c$$

we can find ${\overset{\wedge }{x}}_{3}=1.01$. When there is only defectors and discriminators in the game, there is no stable equilibrium point in the interior of definition domain, i.e., ${\overset{\wedge }{x}}_{3}\notin [0,1]$. However, the equilibrium point will move to $\overset{\wedge }{e}(0,0,1)$ on the edge line of $e_{2}e_{3}$. The equilibrium point tends to move from $e_{2}$ to $e_{3}$ (as in $e_{2}\to e_{3}$ in Figure 1). Under the condition of no cooperator, the defector cannot benefit from the cooperator and will be punished by the discriminator, so the defector will gradually disappear, and the discriminator will win.

In the absence of defector, we have $x_{2}=0$, $R_{1}=R_{3}$, and ${\overset{*}{x}}_{1}+{\overset{*}{x}}_{3}=1$. Thus, the stable equilibrium point is $\overset{*}{e}({\overset{*}{x}}_{1},0,{\overset{*}{x}}_{3})$. According to the equation

$$\begin{array}{l} c(1-p)+(1+p\theta -p-\theta )\pi =(1-p{)}^{2}(1-2\theta )\pi x_{3}+\theta \pi [(1-p{)}^{2}\frac{{\left(x_{3}\right)}^{4}-{\left(x_{3}\right)}^{2(N+1)}}{1-{\left(x_{3}\right)}^{2}}\\ -2(1-p)\frac{{\left(x_{3}\right)}^{2}-{\left(x_{3}\right)}^{N+1}}{1-x_{3}}]-(\pi +\theta \pi -c)(1-p)\frac{{\left(x_{3}\right)}^{2}-{\left(x_{3}\right)}^{N+1}}{1-x_{3}} \end{array},$$

we can obtain the real roots of ${\overset{*}{x}}_{3}$ as −0.92 and 1.03. As $x_{3}\notin [0,1]$, we can conclude that there is no stable equilibrium point in the interior of definition domain when there are only cooperators and discriminators in game. The equilibrium points will tend to either $\overset{*}{e}(1,0,0)$ or $\overset{*}{e}(0,0,1)$. In other words, either the cooperator or the discriminator will win on the edge of $e_{1}e_{3}$. If the cooperator wins, the equilibrium point tends from $e_{3}$ to $e_{1}$ ($e_{3}\to e_{1}$). If the discriminator wins, the equilibrium point tends from $e_{1}$ to $e_{3}$ ($e_{1}\to e_{3}$). When there is no defector, the discriminator does not need to punish the defector and their strategies are consistent. Hence, the points on the edge of $e_{1}e_{3}$ all can be the evolutionary equilibrium.

In the absence of discriminator, $x_{3}=0$, $R_{1}=R_{2}$, the stable equilibrium point is $\overset{\circ }{e}({\overset{\circ }{x}}_{1},{\overset{\circ }{x}}_{2},0)$. If $0<\frac{c-\theta \pi }{(1-2\theta )\pi }<1$, we can find the solution $x_{1}=\frac{c-\theta \pi }{(1-2\theta )\pi }$, and $x_{2}=\frac{\pi -c-\theta \pi }{(1-2\theta )\pi }$. Hence, there is no winner and the stable equilibrium point is $\overset{\circ }{e}(\frac{c-\theta \pi }{(1-2\theta )\pi },\frac{\pi -c-\theta \pi }{(1-2\theta )\pi },0)$. Using the parameter values stated before, the stable equilibrium point is $\overset{\circ }{e}(0.5,0.5,0)$, which means the defector and the cooperator are well-matched in strength. If $\frac{c-\theta \pi }{(1-2\theta )\pi }\leq 0$, we can find the solution $x_{1}=0$, and $x_{2}=1$, which means the defector will win and the stable equilibrium point is $\overset{\circ }{e}(0,1,0)$. If $\frac{c-\theta \pi }{(1-2\theta )\pi }\geq 1$, we can find the solution of $x_{1}=1$ and $x_{2}=0$, which means the cooperator will win and the stable equilibrium point is $\overset{\circ }{e}(1,0,0)$. When there is no discriminator, the equilibrium of the dynamic evolutionary game depends on the value of $\frac{c-\theta \pi }{(1-2\theta )\pi }$. In the static game, we can find that if v>π−c and v−c>0, then mixed Nash equilibrium exists for the game and the dynamic evolutionary game’s equilibrium point is $\overset{\circ }{e}(\frac{c-\theta \pi }{(1-2\theta )\pi },\frac{\pi -c-\theta \pi }{(1-2\theta )\pi },0)$. If v>π−c and v−c≥0, then $\theta >\frac{1}{2}$, which means the importance of cooperation diminishes. The static game’s Nash equilibrium is (defection, defection) and the dynamic evolutionary game’s equilibrium point is $\overset{\circ }{e}(0,1,0)$. If v<π−c and v−c<0, then $\theta <\frac{1}{2}$, which means the cooperation is quite important. The static game’s Nash equilibrium is (cooperation, cooperation) and the dynamic evolutionary game’s equilibrium point is $\overset{\circ }{e}(1,0,0)$.

## 4. Conclusions

Human beings have made great progress. However, we still face many prisoner’s dilemma problems, from big ones like international wars, global trade disputes, climate change, environmental protection, to small ones like personal distrust and discord. It is well known that individual rationality often leads to collective irrationality. We have been searching for solutions to various prisoner’s dilemmas to maintain a cooperative system. Although the science of genetics may reveal that humans are selfish in essence, we need not be too pessimistic. This is because we realize that altruistic behavior is needed to achieve human evolution further. The surplus from altruistic behavior can more than compensate for its cost, which can help achieve evolutionary advantages for human species. Perhaps, as Kant said, we are all rational devil, namely, even devil will be moral or altruistic because of self-interests. As a result, human beings are the only species who can maintain cooperation among non-kin members. Furthermore, our model and analysis show that indirect reciprocity can help maintain such human cooperation. This indicates that if an organization has only cooperators who always cooperate and defectors, such an organization may break down as defectors may exhibit abusive behavior. Therefore, an organization needs to provide incentive as well as deterrence to encourage more altruism behavior.

There are some limitations and potential future research directions with this research. First of all, this paper limits itself in examining human cooperative behavior through the perspective of indirect reciprocity. Thus, one future research direction is to simultaneously examine other closely related perspectives and issues such as the evaluation of human moral beliefs. Second, in our model of indirect reciprocity, a player can observe another player’s choice from the last round but not further. It is quite possible that a player’s choice in the current round is affected by his/her choices from the last few rounds. Hence, it is an important research direction to address this issue. Third, the behavior of indirect reciprocity may itself be impacted by issues like religion and culture. It is worthwhile to incorporate these issues to enrich our model.

### Implication

In the foreseeable future, there will be not only good people (cooperators) but also altruistic punishers (discriminators) and some bad people (defectors). This is also true as far as the game of environmental protection is concerned. It can be seen from our model and analysis that there is no stable evolutionary equilibrium in this game, and it is possible that the proportion of defectors might be the highest. In other words, human endeavors and cooperation toward environmental protection might break down. Therefore, stakeholders like governments, firms, and NGOs should provide incentives and/or penalties, financial or otherwise, to encourage people and organizations to take actions for environmental protection. For example, up to 40% of the food produced for humans in advanced economies like South Korea, the US, and Canada is wasted [22]. To solve this environmental problem, different governments in the US and Canada have offered tax credit for donating excess food inventory. In South Korea, there are also financial penalties for using landfills for food waste. As a result, food waste and its resulted environmental pollution has been reduced significantly.

## Figures and Tables

**Table 1:** Payoff matrix of the game.

Player 1	Player 2	
	Cooperation	Defection
cooperation	π−c; π−c	v−c; v
defection	v; v−c	0; 0
